# Kaempferitrin: A Flavonoid Marker to Distinguish *Camellia oleifera* Honey

**DOI:** 10.3390/nu15020435

**Published:** 2023-01-14

**Authors:** Zhen Li, Qiang Huang, Yu Zheng, Yong Zhang, Bin Liu, Wenkai Shi, Zhijiang Zeng

**Affiliations:** 1 Honeybee Research Institute, Jiangxi Agricultural University, Nanchang 330045, China; 2 Jiangxi Province Key Laboratory of Honeybee Biology and Beekeeping, Jiangxi Agricultural University, Nanchang 330045, China

**Keywords:** *C. oleifera* honey, flavonoid, LC-MS/MS, kaempferitrin, marker

## Abstract

*C. oleifera* is an economically important oilseed crop and medical plant. However, as a characteristic honey resource, the standard protocol used to identify the composition of *C. oleifera* honey has not been established yet. Previously, distinctive flavonoid has been shown as an effective marker to trace the botanical origin of honey. In this study, we examined the flavonoid types in *C. oleifera* honey and nine other monofloral honeys by using liquid chromatography tandem-mass spectrometry (LC-MS/MS) and compared the differences and identified eight distinct flavonoids in *C. oleifera* honey. Then, comparing the 8 flavonoids with the 14 flavonoids common to *C. oleifera* honey and nectar, two distinct flavonoids were identified in *C. oleifera* honey and nectar. Finally, we identified kaempferitrin as the distinct flavonoid marker in *C. oleifera* honey using the degree of influence of the partial least-squares discriminant analysis (PLS-DA) model on *C. oleifera* honey and ployfloral honey.

## 1. Introduction

Honey is a sweet substance that is produced from the nectar of flowers, which is collected by foraging honeybees and mixed with the secreted enzyme and then stored in the hive comb until thoroughly mature [1]. Honeybees can collect nectar from one or more plants to make honey; thus, honey can be classified as monofloral or polyfloral (multi-floral) honey [2]. Honey composition comprises more than 200 different components such as sugar, water, organic acids, minerals, enzymes, proteins, vitamins, ash, polyphenolic compounds, and plant derivatives, etc., [3,4]. The chemical composition, color, and flavor of honey varies depending on the environment, where the plants were grown, and their geographical location, as well as being affected by weather conditions, processing, handling, packaging, and storage time [5].

Honey manifests a variety of medicinal and health benefits as a natural food supplement with a long history of utilization [6]. Honey was first registered as a topical pharmaceutical preparation in Australia in 1999; since then, a range of honey-based products have become available, including sterile Manuka honey ointments and dressings containing honey [6]. Currently, the study of the medical value of medicinal honey extracted from special medicinal plants is a popular research topic [7].

*C. oleifera* is one of the most valuable economic woody crops and medical plant grown in Asia and has been cultivated for more than 2300 years [8]. The main profit driver of growing *C. oleifera* is to obtain the *C. oleifera* seeds and the camellia oil with a highly economical value. *C. oleifera* seeds are abundant in a multitude of bioactive compounds, which have the effect of preventing cardiovascular diseases such as hypertension, coronary heart disease, and atherosclerosis [9]. Camellia oil is acquired from the seeds of *C. oleifera* with a superior color, aroma, and taste, and is commonly regarded as an excellent quality oil because it is easily absorbed and digested by the human body, with a variety of biological activities such as lowering blood pressure, blood lipids, and the softening blood vessels; moreover, the long-term consumption can enhance human immunity, etc., [10]. In addition, camellia oil has powerful antioxidant activity and can serve as a traditional medicine to prevent liver damage and gastrointestinal ulcers caused by oxidative stress [11].

*C. oleifera* honey is derived from nectar collected by honeybees foraging on *C. oleifera* Abel. plants. Previous studies have found that *C. oleifera* honey contains oligosaccharides (manninotriose, raffinose, and stachyose), and it can lead to the death of honeybee larvae and adult worker bees [12,13]. Raffinose and stachyose are classified as raffinose family oligosaccharides (RFOs), one type of prebiotic that has biological functions such as regulating gut flora, preventing inflammatory bowel disease, protecting the liver, and lowering blood sugar and blood lipids, etc., [14,15,16,17]. This implies the promising use of *C. oleifera* honey for the development of potential health promoters and dietary supplements, which is a prioritized direction for subsequent research in our laboratory.

Once the special pharmacological effects of *C. oleifera* honey that are beneficial for health-related functions are proven, then the commercial value of *C. oleifera* honey will increase dramatically. In addition, the wealth of *C. oleifera* growers and beekeepers will also increase owing to the side industry of *C. oleifera* honey. After the commercial value of *C. oleifera* honey has increased, its authenticity is well worth studying [18]. This is because the danger of adulterated honey is not only the use of cheap honey as high-priced honey to achieve a higher value, but, even more so, it will damage the health of consumers. First and foremost, we can initially identify the authenticity of *C. oleifera* honey in terms of its oligosaccharide components and concentration; however, there are limitations to this discriminatory approach. As the RFOs are highly water soluble, it would be feasible to isolate them from *Glycine max*, *Stachys floridana*, and *Stachys sieboldii* and then blend them into common honey [19], posing as *C. oleifera* honey. However, accumulating evidence reveals that the abundant but trace amounts of flavonoids (natural secondary metabolites derived from plants) in honey provide their own distinctive chemical markers [7,20,21]. Hence, the distinct flavonoid markers to identify the authenticity of *C. oleifera* honey is a reliable and novel strategy.

In order to identify the distinctive flavonoid markers in *C. oleifera* honey to facilitate the discrimination of *C. oleifera* honey from other commercial honeys, in the present study, we employed the liquid chromatography tandem-mass spectrometry (LC-MS/MS) technique to identify the types and absolute contents of flavonoid compounds in *C. oleifera* honey, as well as in nine other kinds of monofloral honey and one polyfloral honey. Additionally, the flavonoid species of *C. oleifera* honey with that of *C. oleifera* nectar from the parent plant were identified and applied using partial least-squares discriminant analysis (PLS-DA).

## 2. Materials and Methods

### 2.1. Chemicals and Reagents

Liquid-chromatography mass spectrometry (LC-MS) grade methanol and acetonitrile were purchased from Merck (Darmstadt, Germany). Formic acid (LC-MS grade) was obtained from Sigma–Aldrich (St. Louis, MO, USA). Fructose, glucose, sucrose, melibiose, manninotriose, raffinose, stachyose, and 5-hydroxymethylfurfural (5-HMF) standards (purity > 98%) were obtained from Sigma (St. Louis, MO, USA). Ultra-pure water from MilliQ-system (Millipore Corporation, Billerica, MA, USA) was used throughout the study. Next, 204 flavonoid standards (purity > 98%) were acquired from MedChemExpress Company (Shanghai, China), see details in Supporting Table S1.

### 2.2. Honey and Nectar Samples’ Collection

The mature *C. oleifera* honey (COH) was collected by honeybees (*Apis mellifera*) placed at the *C. oleifera* plantation in Shengqiao Town, Changning City, Hunan Province, and the sample time was from October to November 2021. The *C. oleifera* nectar (CON) was collected using a micro aspirator (Beijing Dalong Xingchuang Experimental Instrument Co., Beijing, China) in Shengqiao Town from three mother plants of *C. oleifera*. Moreover, the sampling schedule was October 2021. *Citrus reticulata* honey (CRH), *Vitex negundo* honey (VNH), *Eriobotrya japonica* honey (EJH), *Litchi chinensis* Sonn honey (LCSH), *Lycium chinense Miller* honey (LCMH), *Ziziphus jujuba* honey (ZJH), *Tilia tuan* honey (TTH), *Brassica napus* honey (BNH), *Robinia pseudoacacia* honey (RPH), nine types of monofloral honey, and one polyfloral honey were provided by Wuhan Baochun Bee Products Company (Wuhan, China). All honey or nectar samples were set up with three biological replicates and stored at −18 °C for subsequent analysis.

### 2.3. Honey and Nectar Preparation

Next, 0.2 g (±0.01 g) of the honey or nectar sample was weighed accurately in a 10 mL capacity centrifuge tube with screw-on caps, and 100 μL of the internal standard working solution at a concentration of 4000 nmol/L and 5000 μL of the 70% methanol solution were added. After 30 min of ultrasound, the samples were centrifuged (12,000 r/min for 5 min at room temperature). Finally, the supernatant was aspirated and filtered through a 0.22 μm filter membrane and 800 μL was transferred to a 1.5 mL injection vial for LC-MS/MS analysis.

### 2.4. Analysis the Chemical Parameters of C. oleifera Honey

#### 2.4.1. Analysis Methods for the Sugar Composition in *C. oleifera* Honey

Thermo ICS 5000 liquid chromatography with an electrochemical detector (Thermo Fisher Technology Inc., Waltham, MA, USA) and a CarboPac PA20 liquid chromatographic column (150 × 3.0 mm, 4 μm) was employed for the analysis of sugar composition in honey samples. The mobile phases were A: H2O, B: 100 mM NaOH; the injection volume was 5 μL, the flow rate was 0.5 mL/min, and the column temperature was 30 °C. Elution gradient: 0.0~9 min, 5% B; 9~20 min, 5~100% B; 20~30 min, 100% B; 30~30.1 min, 100~5% B; 30.1~60 min, 5% B.

#### 2.4.2. Analysis Methods for the Water, Acidity, and 5-HMF Composition in *C. oleifera* Honey

The water content of the honey samples was determined by reading the refractive index of each sample using an Abbe refractometer and brought into the formula: moisture (%) = 100 − [78 + 390.7 × (n − 1.4768)] to calculate the water content. Where n is the actual refractive index of the specimen honey measured at 40 °C.

Next, 4 g of sodium hydroxide was dissolved in 1 L of boiled and cooled water and its concentration was calibrated with potassium hydrogen phthalate (reference reagent) according to the following method: weigh the potassium hydrogen phthalate (reference reagent 0.8~0.9 g (accurate to 0.0002 g) that has been dried in advance at 125 °C, place it in a 250 mL conical flask, dissolve it in 50 mL of boiled and cooled water, and add 2~3 drops of 1% phenolphthalein. Add 2~3 drops of 1% phenolphthalein indicator and titrate with sodium hydroxide solution until the solution is pink, and the end point is that the color does not fade within 10 s.

The concentration of sodium hydroxide standard solution (mol/L) = 0.2042 m/v.

The meaning of the letters in the formula.

c: a concentration of sodium hydroxide standard solution (mol/L).

m: the mass of potassium hydrogen phthalate (g).

v: the volume of sodium hydroxide standard solution consumed at dropwise intervals (mL).

0.2042: a mass of potassium hydrogen phthalate per mL of standard solution of sodium hydroxide [c (NaOH) = 1.000 mol/L] (g).

Weigh 10 g of the honey sample (accurate to 0.001 g). Dissolve in 75 mL of boiled and cooled water, add 2–3 drops of phenolphthalein indicator; titrate with sodium hydroxide standard solution until the solution is pink and does not fade within 10 s as the end point.

The sample acidity (mL/kg) = CV100/m.

v: titration of the volume of sodium hydroxide standard solution was consumed (mL).

c: the molar concentration of sodium hydroxide standard solution (mol/L).

m: the mass of the sample (g).

Note: if the color of honey is too dark, weigh the sample 5 g, or use thymol blue indicator instead of phenolphthalein indicator.

The determination of 5-HMF in *C. oleifera* honey samples was performed on an Agilent 1260 Infinity II liquid chromatography workstation equipped with a Proshell SB C18 column (4.6 × 150 mm, 3.5 μm) (Agilent Technology Inc., Santa Clara, CA, USA). The flow rate was 0.2 m L/min, the column temperature was 30 °C, and the injection volume was 10 μL. Using methanol:water = 8:92 (v:v) as the mobile phase, the detection limit (LOD, S/N = 3) of 5-HMF was obtained as 12 mg/kg at 284 nm UV wavelength.

### 2.5. LC-MS/MS Method for the Determination of Flavonoids in Honey and Nectar

Regarding, the flavonoid standards’ preparation and construction of the standard curve, the 204 flavonoid standards were weighed and prepared into a master batch of 10 mmol/L by methanol–water (70:30) (the concentration of all 204 standards was 10 mmol/L). After that, the master batch was diluted with methanol–water (70:30) and formulated into standard curve working solutions of 0.5 nmol/L, 1 nmol/L, 5 nmol/L, 10 nmol/L, 20 nmol/L, 50 nmol/L, 100 nmol/L, 200 nmol/L, 500 nmol/L, 1000 nmol/L, 2000 nmol/L. Moreover, 100 μL of the internal standard working solution (daidzein) with a concentration of 4000 nmol/L was required and added in each working solution, and the ultimate volume of each working solution was 5 mL. The mass spectral peak intensity data of the corresponding quantitative signals of each concentration standard working solution were acquired. With the concentration ratio of the external standard to the internal standard as the horizontal coordinate and the area ratio of external standard to internal standard ratio as the vertical coordinate, the standard curves of different substances were plotted. The resulting 204 flavonoid standards curve demonstrated a good linearity from 0.5 nmol/L to 200 nmol/L (R^2^ ≥ 0.9900). Results are shown in Supporting Table S2.

### 2.6. LC-MS/MS Analysis

Quantification of flavonoids in honey or nectar was performed on an ultra-performance liquid chromatography system (ExionLC™ AD) coupled with tandem mass spectrometry (QTRAP^®^ 6500+).

Separations were carried out using a Waters ACQUITY UPLC HSS T3 C18 column (1.8 µm, 100 mm × 2.1 mm, Waters, Milford, MA, USA). Analytes were separated using gradient elution with water (containing 0.05%, *v*/*v* formic acid) (A) and acetonitrile (containing 0.05% formic acid, *v*/*v*) (B) at a flow-rate of 0.35 mL/min. The linear gradient elution program was: 0.0~1.0 min, 10~20% B; 1.0~9.0 min, 20~70% B; 9.0~12.5 min, 70~95% B; 12.5~13.5 min, 95% B; 13.5~13.6 min, 95~10% B, 13.6~15 min, 10% B. The column was thermostated at 40 °C and injection volume was 2 μL. The electrospray ionization (ESI) source temperature was 550 °C, while the mass spectrometry voltage was 5500 V in positive ion mode, −4500 V in negative ion mode, and 35 psi of curtain gas (CUR). In the Q-Trap 6500+, each ion pair was scanned for detection based on the optimized declustering potential (DP) and collision energy (CE).

### 2.7. Conversion of Flavonoid Amounts in Honey and Nectar Samples

After substituting the integrated peak area ratio of all identified samples into the linear equation of the standard curve for calculation, and further substituting the calculation formula to calculate, the final data of the content of the substance in the actual sample was attained.

The amounts of flavonoids in the sample (nmol/g) = cV/1,000,000/m.

The meaning of the letters in the formula.

c: the sample concentration value (nmol/L) obtained by substituting the integral peak area ratio of the sample into the standard curve.

V: the volume of the solution used in the extraction (μL).

m: the mass of the sample weighed (g).

### 2.8. Data Processing

MultiQuant 3.0.3 software (AB SCIEX) was used to process the mass spectrometry data, and the retention time and peak shape information of the standards were referenced to guarantee the accuracy of the qualitative quantification by integrating and correcting the mass spectrometry peaks detected in different samples for the analytes. PLS-DA was fulfilled viva Wekemo Bioincloud (Shenzheng, China). Data are expressed as mean ± standard deviation (SD).

## 3. Results and Discussion

### 3.1. Differences in Flavonoid Species among C. oleifera Honey and Nine Kinds of Monofloral Honey

Table 1 shows the basic parameters of *C. oleifera* honey, in which the content of total reducing sugar was 65.71% (fructose content 38.27%, glucose content 27.44%), sucrose content 1.56%, and moisture content 17.62%, all of which were in accordance with European Union honey standards [7]. The harmful hydroxymethyl furfural was not detected in *C. oleifera* honey. Moreover, apart from the common fructose, glucose, and sucrose, *C. oleifera* honey also contains a minor amount of melibiose and manninotriose and a higher content of raffinose and stachyose. These parameters indicate that *C. oleifera* honey is a high-quality honey and has great potential to regulate the gut [19].

**Figure 1:**
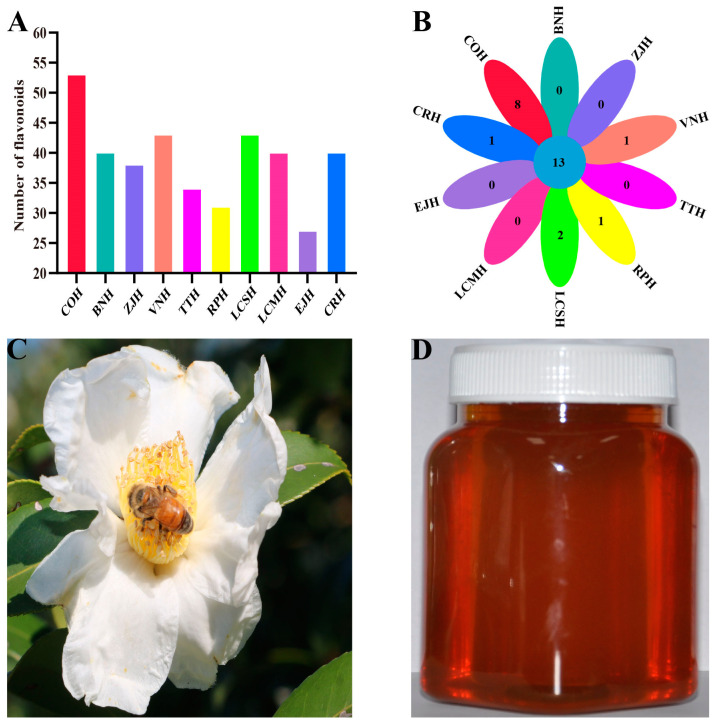
(**A**) Flavonoid species identified in *C. oleifera* honey and 9 monofloral honeys. *C. oleifera* honey (COH), Citrus reticulata honey (CRH), Vitex negundo honey (VNH), Eriobotrya japonica honey (EJH), Litchi chinensis Sonn honey (LCSH), Lycium chinense Miller honey (LCMH), Ziziphus jujuba honey (ZJH), Tilia tuan honey (TTH), Brassica napus honey (BNH), Robinia pseudoacacia honey (RPH). (**B**) Flavonoid species distinctive to *C. oleifera* honey relative to 9 monofloral honeys. (**C**) Honeybee-visited *C. oleifera*. (**D**) *C. oleifera* honey.

There were 54 flavonoids detected in *C. oleifera* honey using LC-MS/MS, which was higher than the remaining nine monofloral honeys (Figure 1A). Likewise, eight distinct flavonoids in *C. oleifera* honey were found, including kaempferitrin, phloretin, acacetin, scutellarein tetramethyl ether, 5,7-dihydroxy-3,4,5-trimethoxyflavone, scutellarin, sinensetin, and tectorigenin (Figure 1B). These differences in the composition and content of flavonoid compounds in different monofloral nectars were predominantly attributed to the pollen of nectar plants [22]. The blossom size of *C. oleifera* is about over six times larger than that of the nectar flowers of common plants and has a large amount of nectar and pollen (Figure 1C). Thus, the honeybees collect more pollen when collecting *C. oleifera* nectar, resulting in a greater variety of flavonoid compounds in *C. oleifera* honey.

### 3.2. Identification of the Distinctive Flavonoid Marker in C. oleifera Honey

**Figure 2:**
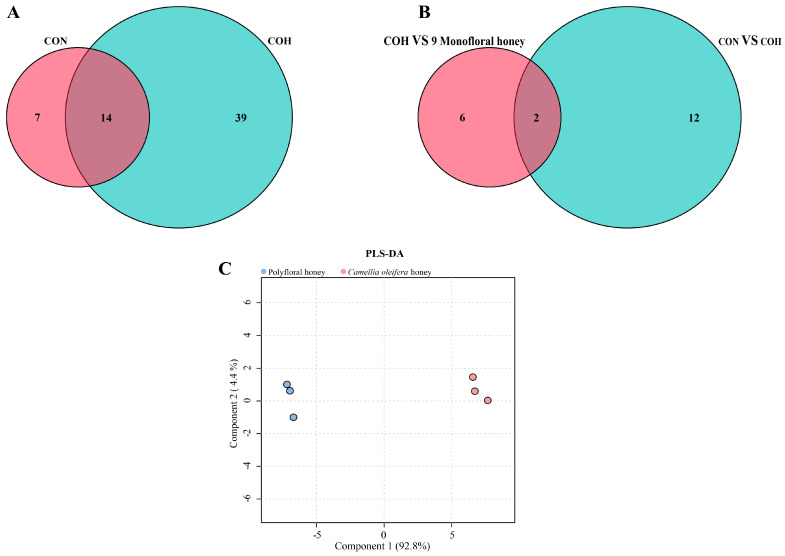
(**A**) The flavonoid species that are shared in *C. oleifera* honey (COH) and *C. oleifera* nectar (CON). (**B**) Flavonoids distinctive to *C. oleifera* honey (COH) and nectar relative to 9 types of monofloral honey. (**C**) PLS-DA of the flavonoid compounds determined by LC-MS/MS of samples of polyfloral and *C. oleifera* honey. The first PLS component explains 92.8% (Component 1) and the second PLS component 4.4% (Component 2) of the variation of the data.

Flavonoids are a large family of phenolic pigments that are natural secondary metabolites derived from plants [23]. Flavonoids in *C. oleifera* honey originate from the nectar of *C. oleifera* and the pollen blended with the nectar. We further identified 21 flavonoid species in *C. oleifera* nectar utilizing LC-MS/MS (Figure 2A). There were 14 flavonoids shared between *C. oleifera* honey and *C. oleifera* nectar (Figure 2A). After further comparison of the 14 flavonoids in common with the 8 flavonoids formerly unique to *C. oleifera* honey relative to the 9 kinds of monofloral honey, two flavonoids (kaempferitrin and phloretin) unique to *C. oleifera* honey and nectar were identified (Figure 2B).

**Figure 3:**
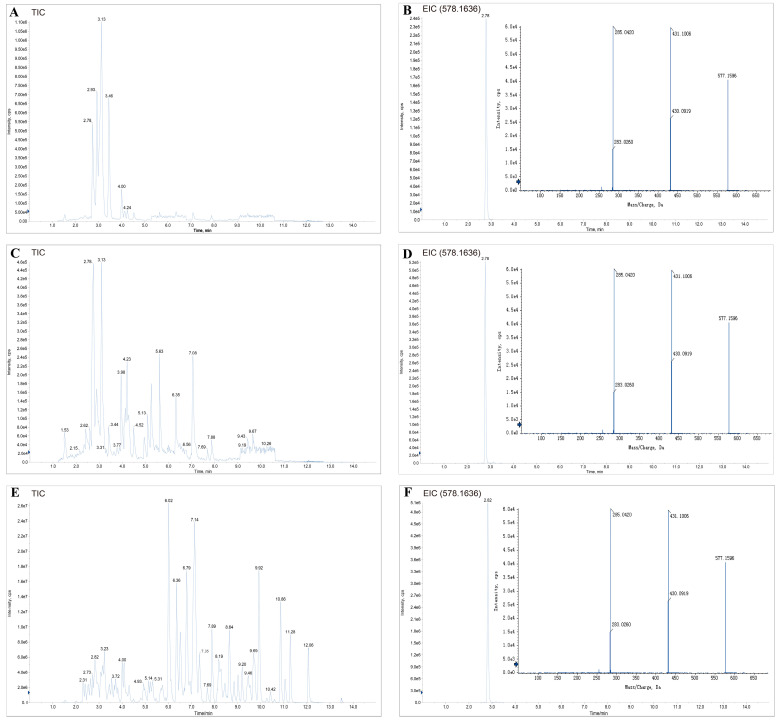
The typical chromatograms of the extracts from the positive/negative mode analyzed by LC-MS/MS: (**A**) Total ion current chromatogram (TIC) of *C. oleifera* nectar. (**B**) Extracted ion chromatogram (EIC) and mass spectrum of kaempferitrin in *C. oleifera* nectar. (**C**) TIC of *C. oleifera* honey. (**D**) EIC and mass spectrum of kaempferitrin in *C. oleifera* honey. (**E**) TIC of kaempferitrin standard. (**F**) EIC and mass spectrum of kaempferitrin standard.

Moreover, we constructed one PLS-DA model based on the types and contents of flavonoids included in *C. oleifera* honey and polyfloral honey (Figure 2C). *C. oleifera* honey and polyfloral honey were well separated in the model, again illustrating that the use of flavonoid components to distinguish honey of different plant origin is a very feasible approach. The variable importance in the projection (VIP) value of kaempferitrin was 1.052, indicating that it had a greater effect on the PLS-DA model between polyfloral and *C. oleifera* honey samples (VIP > 1 is typically regarded as having the great impact on the model) (Supporting Table S3). In contrast, the VIP value of phloretin was 0.968, which had less effect on the PLS-DA model (Supporting Table S3). More importantly, phloretin was present in polyfloral honey as well. Finally, we identified the distinct flavonoid marker in *C. oleifera* honey as kaempferitrin. The chromatogram and mass spectra of kaempferitrin in *C. oleifera* honey and nectar are shown in Figure 3.

### 3.3. Kaempferitrin Quantification and Method Validation

*C. oleifera*, known as the source of camellia oil, is also a versatile plant. For instance, besides being a nutritious edible oil, camellia oil can also be used as anti-rust oil and lubricant for industrial purposes; the pressed *C. oleifera* cake is both a natural fungicide and fertilizer, and the seed peel of *C. oleifera* is a raw material for extracting tannin extract [24]. Kaempferitrin is mainly distributed in the new leaf buds of Camellia sinensis, and it exhibits analgesic, anti-inflammatory, antidiabetic, antitumor, and chemotherapeutic effects, as well as activating insulin signaling [25,26]. Therefore, the examination of kaempferitrin in *C. oleifera* honey not only enables the authentication of *C. oleifera* honey, it also has realistic value for the evaluation of biological activity of *C. oleifera* honey.

Here, a LC-MS/MS method was developed to detect of kaempferitrin: first, a standard curve was established using kaempferitrin standards, and then the intensity of kaempferitrin parent ions in *C. oleifera* honey and nectar samples could be quantified upon this curve and LC-MS/MS. We constructed a standard curve of kaempferitrin with good linearity (regression coefficient = 0.9989) in the range of 0.5–2000 nmol/L as y = 10,383.8424 x + 1676.5839 (Table 2). The limits of detection (LOD, signal to noise ratio = 3) and quantification (LOQ, signal to noise ratio = 10) of kaempferol were 0.07 nmol/kg and 0.25 nmol/kg, respectively (Table 2), and our determination results indicated that the honey and nectar of *C. oleifera* contain 5.98 ± 0.84 and 2.36 ± 0.82 nmol/kg of kaempferitrin, respectively (Table 2). To further validate the method, the RSD of *C. oleifera* honey and nectar were calculated as 1.23% and 1.38%, respectively (Table 2). Overall, the method is sensitive and reliable for the detection of kaempferitrin.

## 4. Conclusions

*C. oleifera* honey is one of the new byproducts of the *C. oleifera* industry, which boosts the income of beekeepers and *C. oleifera* growers and shows huge potential to serve as a medicinal honey. In this experiment, by comparing the flavonoid differences between *C. oleifera* honey and nine monofloral commercial honeys, kaempferitrin was identified as the distinct flavonoid marker of *C. oleifera* honey. An LC-MS/MS method was also developed to detect the content of kaempferitrin in *C. oleifera* honey and nectar samples. The identification of the distinctive flavonoid markers has a practical application for the authentication of *C. oleifera* honey.

## Figures and Tables

**Table 1:** Chemical parameters of *C. oleifera* honey (*n* = 3).

Parameter	Mean ± SD
Fructose, %	38.27 ± 1.06
Glucose, %	27.44 ± 0.71
Sucrose, %	1.56 ± 0.03
Melibiose, %	0.11 ± 0.002
Manninotriose, %	1.44 ± 0.03
Raffinose, %	6.92 ± 0.21
Stachyose, %	7.85 ± 0.21
Water, %	17.62 ± 0.16
Acidity, mL/kg	34.83 ± 0.82
5-HMF, mg/kg	ND

Note: “ND” means not detected.

**Table 2:** Kaempferitrin of standard curve, LOD (nmol/kg), LOQ (nmol/kg) and the content of kaempferitrin in *C. oleifera* honey (nmol/kg) and nectar (nmol/kg).

Compound	Standard Curve	LOD	LOQ	Regression (R^2^)	COH (*n* = 3)		CON (*n* = 3)	
					Content	RSD (%)	Content	RSD (%)
Kaempferitrin	y = 10,383.8424 x + 1676.5839	0.07	0.25	0.9989	5.98 ± 0.84	1.23	2.36 ± 0.82	1.38

## Data Availability

The data in this study are available upon reasonable request.

## Supplementary Materials

The following supporting information can be downloaded at: https://www.mdpi.com/article/10.3390/nu15020435/s1, Table S1: Standard information for 204 flavonoids; Table S2: Standard working curve information for 204 flavonoids; Table S3: Partial least-squares discriminant analysis (PLS-DA) results.
