# Supplementary material for: Kaempferitrin: A Flavonoid Marker to Distinguish Camellia oleifera Honey
Source: Nutrients. 2023 Jan 14;15(2):435. doi: 10.3390/nu15020435 (PMC9867482; doi:10.3390/nu15020435)
Supplement: Supplementary file 1 [file nutrients-15-00435-s001.zip › Table S2.pdf]

Table S2. Standard working curve information for 204 flavonoids.

No.	Abbreviations	RT (min)	Standard curve	Regression (R^2)
1	Flavonoid_01	2.97	y = 3496.6425 x + 512.9095	0.99007
2	Flavonoid_02	2.85	y = 15566.5389 x - 7952.2289	0.99596
3	Flavonoid_03	9.10	y = 1.8179e5 x + 4.5583e4	0.99789
4	Flavonoid_04	4.95	y = 9620.1576 x - 4121.4954	0.99932
5	Flavonoid_05	8.10	y = 2.4702e5 x + 5968.1145	0.99962
6	Flavonoid_06	5.17	y = 7.8518e4 x + 8370.0911	0.99927
7	Flavonoid_07	2.73	y = 258.2069 x - 454.0186	0.99460
8	Flavonoid_08	9.83	y = 6.8149e5 x + 4.3722e5	0.99306
9	Flavonoid_09	4.99	y = 9046.9851 x - 3748.2249	0.99223
10	Flavonoid_10	5.73	y = 21428.8169 x - 153.4533	0.99924
11	Flavonoid_11	2.05	y = 1656.7983 x + 55.3182	0.99895
12	Flavonoid_12	4.12	y = 3.5146e4 x - 193.9155	0.99849
13	Flavonoid_13	2.53	y = 21612.7810 x + 1593.0357	0.99559
14	Flavonoid_14	7.03	y = 4.8686e5 x + 1.8587e5	0.99868
15	Flavonoid_15	3.22	y = 4.2470e4 x + 7952.8170	0.99753
17	Flavonoid_16	9.64	y = 1.4324e5 x + 3.3677e4	0.99979
18	Flavonoid_17	8.21	y = 8.04700e4 x + 4983.8891	0.99991
19	Flavonoid_18	5.40	y = 19255.8306 x + 8918.3731	0.99913
20	Flavonoid_19	13.43	y = 2743.1525 x + 1412.01110	0.99792
21	Flavonoid_20	6.08	y = 19712.9185 x + 1238.1521	0.99845
22	Flavonoid_21	7.81	y = 4.3129e5 x + 6.2743e4	0.99993
23	Flavonoid_22	3.63	y = 5.4161e4 x - 25583.9823	0.99822
24	Flavonoid_23	4.24	y = 18188.4376 x - 8997.4175	0.99903
25	Flavonoid_24	6.85	y = 29093.8624 x + 4773.1787	0.99963
26	Flavonoid_25	3.16	y = 7800.5024 x - 16561.6743	0.99917
27	Flavonoid_26	5.93	y = 1.0968e5 x + 5697.1219	0.99967
28	Flavonoid_27	8.27	y = 7831.4471 x - 8019.8193	0.99894
29	Flavonoid_28	2.85	y = 4213.0199 x + 1709.4104	0.99620
30	Flavonoid_29	5.59	y = 3.1863e4 x - 1500.0051	0.99924
31	Flavonoid_30	5.68	y = 4.9050e4 x + 3632.9299	0.99966
32	Flavonoid_31	3.41	y = 3.1760e4 x - 13073.3038	0.99622
33	Flavonoid_32	2.59	y = 4095.6830 x + 3459.5859	0.99648
34	Flavonoid_33	3.55	y = 5608.1314 x - 4688.8779	0.99904
35	Flavonoid_34	8.38	y = 3.7136e4 x + 18451.6057	0.99922
36	Flavonoid_35	3.10	y = 668.9319 x + 6033.7250	0.99919
37	Flavonoid_36	6.42	y = 5190.6629 x - 2131.6717	0.99433
38	Flavonoid_37	7.24	y = 14140.9256 x - 2421.0231	0.99836
39	Flavonoid_38	10.18	y = 15218.5618 x + 1937.5978	0.99923
40	Flavonoid_39	6.73	y = 2.6073e5 x + 3.9574e4	0.99950
41	Flavonoid_40	2.64	y = 4287.1949 x - 331.8465	0.99729

Continue for Table S2

No.	Abbreviations	RT (min)	Standard curve	Regression (R^2)
42	Flavonoid_42	2.91	y = 25483.9708 x - 11416.3105	0.99713
43	Flavonoid_43	5.09	y = 1552.7710 x + 952.1216	0.99882
44	Flavonoid_44	6.74	y = 26114.5130 x + 2963.7406	0.99979
45	Flavonoid_45	4.16	y = 209.8973 x + 2426.8721	0.99805
46	Flavonoid_46	6.49	y = 8.3511e4 x + 824.9585	0.99984
47	Flavonoid_47	8.18	y = 4.02990e4 x + 17623.1355	0.99906
48	Flavonoid_48	2.97	y = 16247.2283 x - 1296.1706	0.99980
49	Flavonoid_49	3.52	y = 3023.0384 x + 5882.7964	0.99507
50	Flavonoid_50	4.19	y = 18298.5057 x + 3741.6848	0.99948
51	Flavonoid_51	2.37	y = 15023.4567 x + 2622.0209	0.99943
52	Flavonoid_52	2.77	y = 17028.0635 x - 2318.0944	0.99930
53	Flavonoid_53	8.46	y = 17251.4617 x + 462.6047	0.99851
54	Flavonoid_54	2.80	y = 4.2203e4 x + 845.4219	0.99987
55	Flavonoid_55	2.21	y = 1442.9940 x + 389.6780	0.99934
56	Flavonoid_56	3.13	y = 6293.8263 x - 22503.4148	0.99804
57	Flavonoid_57	2.72	y = 4.0151e4 x - 11812.4468	0.99699
58	Flavonoid_58	5.26	y = 3.4376e4 x + 1499.0156	0.99855
59	Flavonoid_59	5.08	y = 4642.13674 x + 3737.0140	0.99827
60	Flavonoid_60	10.97	y = 7.0704e4 x + 12165.5237	0.99860
61	Flavonoid_61	6.93	y = 6722.5720 x - 32.1165	0.99988
62	Flavonoid_62	9.80	y = 16841.9008x - 616.9100	0.99970
63	Flavonoid_63	5.09	y = 4987.6840 x + 10571.6284	0.99745
64	Flavonoid_64	3.71	y = 4277.3330 x + 11537.6571	0.99542
65	Flavonoid_65	3.35	y = 8462.7319 x - 2785.6658	0.99181
66	Flavonoid_66	2.30	y = 3.7444e4 x + 10111.0570	0.99969
67	Flavonoid_67	3.49	y = 8525.5565 x + 28036.6841	0.99271
68	Flavonoid_68	2.30	y = 14312.7021 x - 487.9446	0.99977
69	Flavonoid_69	2.87	y = 24506.1452 x + 4609.2442	0.99897
70	Flavonoid_70	7.83	y = 15622.5086 x + 4433.0085	0.99851
71	Flavonoid_71	7.20	y = 1762.7511 x + 444.8389	0.99804
72	Flavonoid_72	4.93	y = 12713.8853 x - 756.1911	0.99986
73	Flavonoid_73	2.97	y = 2886.7319 x + 11091.6526	0.99707
74	Flavonoid_74	2.47	y = 4738.7632 x - 2050.6350	0.99502
75	Flavonoid_75	8.06	y = 14435.7306 x + 3782.6976	0.99939
76	Flavonoid_76	7.01	y = 4.4036e4 x + 1133.7363	0.99993
77	Flavonoid_77	3.70	y = 72.3503 x - 1078.2661	0.99338
78	Flavonoid_78	2.71	y = 17532.8915 x - 1799.4911	0.99780
79	Flavonoid_79	2.80	y = 10061.7419 x + 923.3292	0.99535
80	Flavonoid_80	2.56	y = 789.1705 x + 418.2928	0.99061
81	Flavonoid_81	3.95	y = 3.2862e4 x + 8.3014e4	0.99729
82	Flavonoid_82	6.97	y = 3.9732e4 x + 2404.5215	0.99984

Continue for Table S2

No.	Abbreviations	RT (min)	Standard curve	Regression (R^2)
83	Flavonoid_83	3.88	y = 87.6350 x + 211.7865	0.99174
84	Flavonoid_84	1.97	y = 3591.0249 x + 4181.9997	0.99879
85	Flavonoid_85	3.89	y = 147.0537 x + 5794.8446	0.99421
86	Flavonoid_86	3.07	y = 4.4075e4 x - 17715.6410	0.99611
87	Flavonoid_87	9.61	y = 3.9667e4 x + 22551.2305	0.99916
88	Flavonoid_88	8.57	y = 1.6049e5 x + 10052.0355	0.99974
89	Flavonoid_89	4.68	y = 3234.2959 x - 321.5477	0.99921
90	Flavonoid_90	2.86	y = 25915.2794 x + 2751.5377	0.99813
91	Flavonoid_91	11.01	y = 29016.1215 x + 136.9777	0.99989
92	Flavonoid_92	3.47	y = 16340.2616 x - 3.9525e4	0.99813
93	Flavonoid_93	7.63	y = 5.9771e4 x + 9815.2687	0.99903
94	Flavonoid_94	3.78	y = 12.8920 x - 134.0203	0.99025
95	Flavonoid_95	4.27	y = 4.6110e4 x + 392.5080	0.99987
96	Flavonoid_96	2.78	y = 142.9436 x + 2283.4508	0.99286
97	Flavonoid_97	9.57	y = 24333.3338 x + 2883.8791	0.99915
98	Flavonoid_98	2.83	y = 38.7985 x + 1611.9214	0.99545
99	Flavonoid_99	7.02	y = 6.2917e4 x + 12334.0999	0.99954
100	Flavonoid_100	8.55	y = 4.2756e4 x + 1737.9514	0.99995
101	Flavonoid_101	3.12	y = 14216.8125 x - 587.2457	0.99899
102	Flavonoid_102	3.67	y = 177.1754 x + 49.4876	0.99772
103	Flavonoid_103	7.08	y = 6.8445e5 x + 8.0731e4	0.99853
104	Flavonoid_104	8.33	y = 29974.3474 x + 1419.3770	0.99979
105	Flavonoid_105	10.76	y = 4.3173e5 x + 1.6250e5	0.99770
106	Flavonoid_106	2.60	y = 7746.6929 x - 1832.8736	0.99957
107	Flavonoid_107	6.89	y = 2745.1701 x + 349.6255	0.99948
108	Flavonoid_108	7.13	y = 4479.6896 x + 1063.0917	0.99864
109	Flavonoid_109	9.42	y = 9848.8467 x + 29416.7906	0.99644
110	Flavonoid_110	7.27	y = 1.0256e5 x + 15868.5305	0.99986
111	Flavonoid_111	6.70	y = 4.6085e5 x + 9.0346e4	0.99771
112	Flavonoid_112	4.88	y = 6261.7302 x + 10223.4224	0.99744
113	Flavonoid_113	2.64	y = 553.4538 x - 3904.9460	0.99182
114	Flavonoid_114	3.28	y = 5187.8839 x - 62.5617	0.99864
115	Flavonoid_115	3.11	y = 17884.2952 x - 7175.7811	0.99512
116	Flavonoid_116	11.20	y = 1.9620e5 x + 5.7424e4	0.99924
117	Flavonoid_117	3.24	y = 6253.9626 x - 1655.7401	0.99929
118	Flavonoid_118	2.94	y = 24285.2218 x - 5030.4943	0.99884
119	Flavonoid_119	3.07	y = 26569.6166 x + 5206.1622	0.99800
120	Flavonoid_120	9.87	y = 3.3749e4 x - 854.9501	0.99958
121	Flavonoid_121	8.04	y = 3.4053e4 x + 1881.2384	0.99998
122	Flavonoid_122	2.66	y = 2680.2740 x + 1584.7526	0.99873
123	Flavonoid_123	2.84	y = 17627.9112 x - 9.6075e4	0.99000

Continue for Table S2

No.	Abbreviations	RT (min)	Standard curve	Regression (R^2)
124	Flavonoid_124	3.68	y = 5751.2597 x - 917.6386	0.99971
125	Flavonoid_125	5.63	y = 4.1680e4 x + 6499.9719	0.99932
126	Flavonoid_126	4.15	y = 4550.5671 x - 1734.3066	0.99933
127	Flavonoid_127	3.97	y = 0.0366 x + 0.0071	0.99949
128	Flavonoid_128	9.31	y = 7.1911e4 x + 3.0214e4	0.99918
129	Flavonoid_129	8.76	y = 5.3015e4 x + 4159.3912	0.99959
130	Flavonoid_130	9.46	y = 4.5846e4 x + 6754.1625	0.99968
131	Flavonoid_131	7.28	y = 5.4005e4 x + 3323.0636	0.99970
132	Flavonoid_132	5.21	y = 6070.8165 x + 757.1672	0.99984
133	Flavonoid_133	12.00	y = 1.9707e5 x + 2.1048e5	0.99948
134	Flavonoid_134	7.31	y = 13086.4389 x + 7698.2012	0.99944
135	Flavonoid_135	3.88	y = 221.9623 x - 5295.0801	0.99470
136	Flavonoid_136	4.75	y = 14406.2430 x + 1272.7275	0.99992
137	Flavonoid_137	3.97	y = 5.0752e4 x - 5741.6665	0.99954
138	Flavonoid_138	2.48	y = 3.0130e4 x - 9294.5188	0.99932
139	Flavonoid_139	3.61	y = 10291.9122 x - 1862.6917	0.99948
140	Flavonoid_140	6.82	y = 28042.8267 x + 2792.4557	0.99976
141	Flavonoid_141	4.45	y = 13145.8754 x - 2461.0180	0.99910
142	Flavonoid_142	10.33	y = 3.7666e4 x + 18470.1659	0.99921
143	Flavonoid_143	5.28	y = 3.4733e4 x - 327.8250	0.99944
144	Flavonoid_144	6.62	y = 5.2859e4 x + 18532.5754	0.99870
145	Flavonoid_145	2.27	y = 568.7046 x + 34.0869	0.99888
146	Flavonoid_146	4.09	y = 16.3733 x + 181.8778	0.99858
147	Flavonoid_147	2.45	y = 2459.5207 x - 1253.0410	0.99937
148	Flavonoid_148	7.10	y = 9.3466e4 x + 2201.7367	0.99995
149	Flavonoid_149	6.58	y = 13943.2656 x + 438.9954	0.99994
150	Flavonoid_150	2.91	y = 4963.9097 x - 1886.7672	0.99644
151	Flavonoid_151	6.30	y = 7.5518e5 x + 8.6712e4	0.99941
152	Flavonoid_152	3.62	y = 202.2768 x + 243.7588	0.99171
153	Flavonoid_153	2.62	y = 400.6695 x + 176.4194	0.99492
154	Flavonoid_154	2.78	y = 5.6954e4 x - 12486.3815	0.99727
155	Flavonoid_155	2.67	y = 2803.4094 x + 330.4011	0.99967
156	Flavonoid_156	4.27	y = 1184.1218 x - 313.9026	0.99505
157	Flavonoid_157	2.37	y = 25379.6696 x - 1483.7026	0.99987
158	Flavonoid_158	7.06	y = 13003.7984 x + 121.1500	0.99977
159	Flavonoid_159	10.51	y = 11171.7878 x + 3435.8948	0.99851
160	Flavonoid_160	3.14	y = 26692.1834 x + 1095.2639	0.99891
161	Flavonoid_161	3.05	y = 3.7054e4 x - 4780.7855	0.99920
162	Flavonoid_162	9.58	y = 1.9862e5 x + 11346.8220	0.99970
163	Flavonoid_163	6.87	y = 6094.8884 x + 2582.3638	0.99949
164	Flavonoid_164	6.43	y = 1.7239e5 x + 7.5198e5	0.99278

Continue for Table S2

No.	Abbreviations	RT (min)	Standard curve	Regression (R^2)
165	Flavonoid_165	3.20	y = 4.6409e4 x + 3024.2730	0.99942
166	Flavonoid_166	7.14	y = 5.1250e4 x + 14648.2227	0.99953
167	Flavonoid_167	3.03	y = 2756.4724 x + 1589.6321	0.99470
168	Flavonoid_168	5.96	y = 9.0055e5 x + 4.6388e5	0.99618
169	Flavonoid_169	2.70	y = 18533.0814 x - 1994.8434	0.99962
170	Flavonoid_170	8.93	y = 9817.4055 x + 986.4704	0.99924
171	Flavonoid_171	5.84	y = 16500.9343 x + 8401.6272	0.99918
172	Flavonoid_172	2.82	y = 10383.8424 x + 1676.5839	0.99892
173	Flavonoid_173	8.54	y = 1.8127e5 x + 6.1977e4	0.99807
174	Flavonoid_174	8.77	y = 3.0902e4 x + 5192.9354	0.99978
175	Flavonoid_175	3.42	y = 3.3583e4 x + 3299.0459	0.99788
176	Flavonoid_176	3.20	y = 2477.6624 x + 9678.2655	0.99897
177	Flavonoid_177	4.87	y = 4.7778e4 x - 9920.0653	0.99930
178	Flavonoid_178	2.81	y = 2884.4000 x - 13159.8870	0.99424
179	Flavonoid_179	4.05	y = 5.5284e4 x - 16385.3707	0.99861
180	Flavonoid_180	3.15	y = 3.2279e4 x - 6831.9510	0.99765
181	Flavonoid_181	3.45	y = 4471.4799 x - 3738.1077	0.99815
182	Flavonoid_182	3.51	y = 21986.6748 x + 9881.9627	0.99944
183	Flavonoid_183	4.03	y = 27073.6971 x + 7065.4897	0.99891
184	Flavonoid_184	2.57	y = 3410.5695 x - 1499.5637	0.99867
185	Flavonoid_185	6.45	y = 1.4866e5 x + 28357.5354	0.99985
186	Flavonoid_186	7.30	y = 12965.8656 x + 2646.0875	0.99887
187	Flavonoid_187	3.51	y = 3.5976e4 x - 8072.6614	0.99900
188	Flavonoid_188	4.24	y = 17620.5564 x + 4459.8258	0.99964
189	Flavonoid_189	2.48	y = 13417.3324 x - 4.6589e4	0.99504
190	Flavonoid_190	3.73	y = 12762.3978 x + 9359.7886	0.99850
191	Flavonoid_191	4.94	y = 16752.6144 x + 4002.3187	0.99945
192	Flavonoid_192	2.61	y = 20236.5879 x - 10551.1954	0.99251
193	Flavonoid_193	6.04	y = 29989.4143 x + 10415.5252	0.99949
194	Flavonoid_194	2.81	y = 1262.6683 x + 3665.5675	0.99603
195	Flavonoid_195	1.52	y = 4516.0333 x - 759.3914	0.99918
196	Flavonoid_196	3.69	y = 5.6989e4 x + 2839.3927	0.99953
197	Flavonoid_197	3.10	y = 29069.3649 x - 4466.4270	0.99574
198	Flavonoid_198	6.74	y = 6.6015e4 x + 20497.3341	0.99762
199	Flavonoid_199	7.30	y = 812.0470 x - 2050.1988	0.99902
200	Flavonoid_200	9.37	y = 8.7260e4 x + 16781.7642	0.99990
201	Flavonoid_201	3.58	y = 28836.7559 x - 6991.5993	0.99802
202	Flavonoid_202	3.11	y = 5261.1022 x - 4988.7514	0.99246
203	Flavonoid_203	3.79	y = 2306.0507 x - 3624.5203	0.99957
204	Flavonoid_204	3.79	y = 35.2656 x - 581.2067	0.99245
