# Supplementary material for: Kaempferitrin: A Flavonoid Marker to Distinguish Camellia oleifera Honey
Source: Nutrients. 2023 Jan 14;15(2):435. doi: 10.3390/nu15020435 (PMC9867482; doi:10.3390/nu15020435)
Supplement: Supplementary file 1 [file nutrients-15-00435-s001.zip › Table S3.pdf]

Table S3. Partial least-squares discriminant analysis (PLS-DA) results.

No.	Compounds	VIP	T. stat	P. value	FDR
1	Kaempferitrin	1.052	-29.600	3.29e-4	6.37e-4
2	Phloretin	0.968	4.631	3.97e-2	4.49e-2
3	Benzylideneacetophenone	1.053	-42.172	2.83e-5	9.31e-5
4	Naringenin chalcone	1.053	43.423	9.80e-5	2.56e-4
5	Narirutin	1.054	-160.400	1.53e-7	2.29e-6
6	Hesperetin	1.052	-31.890	6.92e-4	1.09e-3
7	Alpinetin	1.050	23.945	1.40e-3	1.94e-3
8	Pinocembrin	1.052	34.742	1.47e-5	6.32e-5
9	Hesperidin	1.048	-18.341	2.35e-3	2.88e-3
10	Eriocitrin	1.049	-20.074	1.43e-3	1.94e-3
11	Isosakuranetin	0.749	-2.022	0.14	0.13
12	Liquiritigenin	1.054	-82.257	1.54e-6	1.03e-5
13	Eriodictyol	0.232	0.452	0.676	0.688
14	Taxifolin	1.048	18.913	1.74e-4	3.73e-4
15	Dihydrokaempferol	1.050	23.694	7.18e-5	2.06e-4
16	Vitexin	1.045	15.037	1.65e-3	2.20e-3
17	Isoorientin	1.039	11.548	2.03e-3	2.54e-3
18	Orientin	1.054	-207.160	2.22e-5	8.34e-5
19	Schaftoside	1.027	-8.586	1.75e-3	2.28e-3
20	Sinensetin	1.053	-40.715	7.56e-5	2.06e-4
21	Scutellarin	1.053	-51.971	1.02e-4	2.56e-4
22	4,5-Dihydroxyflavone	1.054	66.881	2.83e-05	9.31e-5
23	5,7-Dihydroxy-3,4,5-trimethoxyflavone	0.508	-1.101	0.385	0.412
24	Scutellarein tetramethyl ether	1.031	-9.357	7.37e-3	8.67e-3
25	Acacetin	1.052	-29.785	5.32e-4	9.07e-4
26	Narcissin	1.054	90.794	4.60e-7	4.05e-6
27	Diosmin	0.143	0.273	0.80	0.80
28	Galangin	1.045	15.063	1.16e-3	1.70e-3
29	Nobiletin	1.054	-86.921	8.20e-7	6.15e-6
30	Chrysin	1.052	36.758	4.05e-4	7.36e-4
31	Diosmetin	1.053	49.631	1.35e-4	3.00e-4
32	Jaceosidin	1.024	-8.226	8.66e-3	0.01
33	Apigenin	0.243	-0.474	0.66	0.69
34	Sakuranetin	1.051	-26.202	4.53e-4	8.00e-4
35	Tangeretin	1.052	-34.087	2.20e-4	4.55e-4
36	Nicotiflorin	1.052	35.974	1.91e-5	7.65e-5
37	Luteolin	1.042	-12.999	7.99e-4	1.23e-3
38	Tectochrysin	1.048	18.185	3.19e-4	6.37e-4

Continue for Table S3

No.	Compounds	VIP	T. stat	P. value	FDR
39	Isorhamnetin-3-O-glucoside	1.054	135.690	4.73e-7	4.05e-6
40	Isorhamnetin-3-O-neohespeidoside	1.054	258.610	3.87e-9	2.32e-7
41	3,7-Di-O-methylquercetin	1.054	166.980	7.99e-9	2.40e-7
42	Tiliroside	1.053	-37.463	1.23e-4	2.90e-4
43	Spiraeoside	1.054	134.540	2.03e-7	2.44e-6
44	Laricitrin	0.362	-0.730	0.51	0.53
45	Astragalin	1.042	13.011	1.95e-3	2.50e-3
46	Baimaside	1.054	85.158	7.19e-5	2.06e-4
47	Quercetin	1.009	6.608	4.18e-3	5.01e-3
48	Kaempferol-3-neohesperidoside	1.044	14.667	1.26e-4	2.90e-4
49	Rutin	1.053	60.225	8.52e-6	4.82e-5
50	Robinin	1.052	33.550	6.34e-4	1.03e-3
51	Afzelin	1.041	-12.702	3.67e-4	6.89e-4
52	Kaempferol	1.049	20.290	5.44e-4	9.07e-4
53	Genistein	1.053	-60.036	9.91e-6	4.95e-5
54	Tectorigenin	1.054	-119.090	3.40e-8	6.81e-7
55	Formononetin	1.053	-43.176	1.46e-5	6.32e-5
56	Daidzein	0.921	-3.601	0.07	0.08
57	Calycosin	1.053	-60.430	8.83e-6	4.82e-5
58	Isomangiferin	1.053	-50.090	2.95e-5	9.31e-5
